# Different Cognitive Profiles of Patients with Severe Aphasia

**DOI:** 10.1155/2017/3875954

**Published:** 2017-05-29

**Authors:** Chiara Valeria Marinelli, Simona Spaccavento, Angela Craca, Paola Marangolo, Paola Angelelli

**Affiliations:** ^1^Lab of Applied Psychology and Intervention, Department of History Society and Human Studies, University of Salento, Lecce, Italy; ^2^IRCCS Foundation Santa Lucia, Rome, Italy; ^3^Neurorehabilitation Unit, Department of Humanities Studies, ICS Maugeri SPA SB, IRCCS Institute of Cassano Murge, Bari, Italy; ^4^Department of Humanities Studies, University of Napoli Federico II, Napoli, Italy

## Abstract

Cognitive dysfunction frequently occurs in aphasic patients and primarily compromises linguistic skills. However, patients suffering from severe aphasia show heterogeneous performance in basic cognition. Our aim was to characterize the cognitive profiles of patients with severe aphasia and to determine whether they also differ as to residual linguistic abilities. We examined 189 patients with severe aphasia with standard language tests and with the CoBaGA (Cognitive Test Battery for Global Aphasia), a battery of nonverbal tests that assesses a wide range of cognitive domains such as attention, executive functions, intelligence, memory, visual-auditory recognition, and visual-spatial abilities. Twenty patients were also followed longitudinally in order to assess their improvement in cognitive skills after speech therapy. Three different subgroups of patients with different types and severity of cognitive impairment were evidenced. Subgroups differed as to residual linguistic skills, in particular comprehension and reading-writing abilities. Attention, reasoning, and executive functions improved after language rehabilitation. This study highlights the importance of an extensive evaluation of cognitive functions in patients with severe aphasia.

## 1. Introduction

Formerly, aphasia was considered exclusively as a linguistic deficit [1]. However, it is difficult to explain the variability of patients with aphasia if only linguistic factors are considered [2, 3]. McNeil and Kimelman [4] suggested that other cognitive impairments in addition to linguistic deficits might compromise the communicative skills of aphasic patients. As the association between language function and cognition is stronger in more severe aphasic conditions [5], there is now greater interest in studying the neuropsychological deficits associated with linguistic impairment in severe aphasics.

Cognitive impairments have frequently been observed in patients with aphasia [6]. A recent review by Fonseca et al. [7] of 47 studies (with a total of 1710 aphasic patients) found that 61.3% of studies showed that patients with aphasia following a stroke tend to obtain lower scores than healthy subjects on most nonverbal cognitive tests. Several studies highlighted the presence of memory deficits (e.g., [8]), attention (e.g., [9]), recognition abilities (e.g., [10]), logic skills (e.g., [11]), and executive functions (e.g., [12]) in aphasic patients.

According to some authors, the occurrence of other cognitive deficits in association with language impairment can seriously worsen the symptomatology of aphasia [13] and may influence the efficacy of rehabilitative training [13, 14]. In fact, it has been found that patients with aphasia and concomitant cognitive deficits benefit less from speech therapy than patients without cognitive deficits [15, 16]. On the other hand, a high level of cognitive abilities predicts better and faster recovery of linguistic abilities [17]. Furthermore, patients with persisting aphasia were found to be more cognitively impaired and severe cognitive impairment is associated with poor functional outcome [18]. Many studies investigating patients with aphasia examined the integrity of a single cognitive function and its relationship to linguistic abilities; by contrast, studies investigating a wider range of cognitive domains (thus providing a profile of the cognitive impairment) are rare. Recently, some studies (e.g., [5, 19, 20]) tried to examine the cognitive deficit in aphasia; however, the question was largely unresolved because of the small number of subjects in the sample, the heterogeneity of clinical types of aphasia, and the need for verbal responses in most nonlinguistic cognitive tests. In fact, it is not easy to test the cognitive abilities of aphasic individuals because neuropsychological tests have a linguistic mediation and are therefore inappropriate for use with this population. Moreover, many tests are too complex; thus, patients with severe aphasia show an invariant profile with very low accuracy (floor effect).

For this reason, some authors have introduced simple nonverbal test batteries for assessing the cognitive abilities of aphasic patients. For example, Kalbe et al. [13] developed the Aphasia Check List (ACL). This nonverbal test battery allows assessing linguistic abilities as well as visual memory, selective attention, and logical reasoning. The authors found that 94% of the 154 patients examined (with moderate to severe aphasia) presented a deficit in at least one of the cognitive functions investigated. According to this study, linguistic performance correlates with memory, attention, and reasoning. El Hachioui et al. [18] used a nonlinguistic cognitive examination to test 147 aphasic patients. It included abstract reasoning, visual memory, visual perception and construction, and executive functioning. The authors found that 88% of the patients were impaired in at least one nonlinguistic cognitive domain after three months and 80% after one year. Impairment of visual memory was most frequent at three months and one year. Impairment of visual perception and construction was least common, and performance on this task was adequate after one year. Similarly, Kauhanen et al. [21] investigated visual memory, problem solving, and visual-constructive abilities in 31 aphasic patients (with different types of aphasia) using some nonverbal tests derived from standard neuropsychological batteries. The authors found that the aphasic patients' performance (but not that of left brain-damaged patients without aphasia) was impaired in all functions investigated also when patients with severe comprehension deficits (global, Wernicke's and transcortical sensory aphasia) were excluded from the sample. This finding was replicated three and 12 months after the stroke. Moreover, all patients suffering from moderate/severe aphasia obtained lower scores on all of the nonverbal cognitive tests compared to patients with mild aphasia.

Helm-Estabrooks [6] also examined the cognitive profile of 13 patients with moderate to severe aphasia using the Cognitive Linguistic Quick Test (CLQT; [22]), which assesses the integrity of language as well as executive functions, attention, memory, and visual-spatial abilities. The author found that the patients' performances were extremely different on the various cognitive tests. Helm-Estabrooks also found that the level of the cognitive deficit is not usually linked to the severity of aphasia. The absence of a correlation between linguistic deficit and cognitive impairment was also demonstrated in a preliminary study [23] of 34 aphasic patients, none of whom had global aphasia. Helm-Estabrooks' studies demonstrate the importance of carrying out a comprehensive cognitive assessment, because the integrity of nonlinguistic abilities cannot be estimated according to the severity of aphasia.

Van Mourik et al. [24] studied the cognitive abilities of patients with global aphasia. The authors examined the performance of 17 patients with global aphasia using the GANBA (Global Aphasic Neuropsychological Battery), which includes nonverbal tests aimed at assessing auditory comprehension and the following cognitive functions: attention/concentration, memory, intelligence, and visual and auditory nonverbal recognition. The authors reported that the three subgroups of patients could be separated into those with global aphasia who had different cognitive profiles: (i) the first group had almost spared cognitive functions and thus required a neurolinguistic treatment; (ii) the second group suffered from a selective deficit in attention and visual-auditory recognition; (iii) and the third group displayed severe cognitive impairments that made the rehabilitative treatment impossible. Patients belonging to the last group had no possibility of communicating and could only express their emotions with facial expressions. Also in this study, the degree of cognitive impairment was independent from the language dysfunction, at least regarding auditory comprehension.

Hinckley and Nash [25] replicated the study of Van Mourik et al. [24] using the GANBA in four patients with mild aphasia, 21 patients with moderate aphasia, and four patients with severe aphasia. The authors found that selective attention, auditory recognition, and memory abilities were related to the severity of aphasia. This finding is in contrast with the results of Van Mourik et al. [24] and Helm-Estabrooks et al. [22, 23].

In summary, according to the studies mentioned above, most patients with aphasia also suffer from other neuropsychological disorders [13]. Moreover, the population of patients with aphasia seems to be extremely heterogeneous as to type and severity of cognitive dysfunctions [6, 24]. However, only one study [24] extended the investigation to a wider range of cognitive domains and identified different subgroups of patients on the basis of their cognitive profile. Unfortunately, this latter study was based exclusively on a qualitative evaluation of cognitive test performances. On the basis of our knowledge, no studies have investigated whether different cognitive profiles are associated with a deficit in a specific linguistic domain. Moreover, the above-mentioned studies reported discordant results about the relationship between cognitive and linguistic dysfunctions. Some studies declared that cognitive and linguistic impairments were independent, and others found that cognitive deficits depended on the severity of the linguistic disorder. These discordant results might also be due to the selection of a small sample with different aphasic disturbances. The extreme variability of cognitive abilities among patients with aphasia necessarily requires the use of large samples [25] to allow the generalization of results.

The first aim of the present study was to evaluate the existence of different profiles of cognitive impairment in a large sample of patients with severe aphasia based on the severity and type of cognitive deficits (study 1). The cognitive abilities studied were attention, executive functions, logical reasoning, visual-spatial ability, memory, and visual and auditory recognition. The integrity of these functions was evaluated with a battery of simple tests that do not require verbal responses and are, thus, suitable for patients with severe aphasia. We were also interested in verifying whether patients with different profiles of cognitive deficits also differ in terms of their residual linguistic skills.

The second aim of the present study was to examine whether speech therapy also improves cognitive skills and which cognitive skill is predictive of greater language recovery (study 2). For this purpose, we examined a group of patients longitudinally pre and post treatment.

## 2. Study 1

In this study, we assessed the existence of different profiles of cognitive impairment in patients with severe aphasia and the relationship between cognitive and linguistic skills. The linguistic skills investigated were oral and written comprehension, naming, reading-spelling, and repetition.

## 3. Participants

One hundred eighty-nine patients (111 males and 78 females), mean age 66 years (SD: ±11.6), were examined 126 days (SD: ±180) after a stroke. Mean educational level was 6.3 years (SD: ±4). The inclusion criteria were the presence of a selective lesion of the left cerebral hemisphere and severe aphasia. In particular, only patients with a score on the Token Test ([26]; see 2.2a) less than 15 (mean accuracy = 6.4; SD: ±4.8) were included. All subjects were Italian and have global aphasia. Patients were excluded if they had bilateral lesions, previous stroke, previous drug abuse, and a positive history of psychiatric disorders or dementia (OMS, 1994).

The performance of patients with aphasia was compared to the performance of healthy subjects paired for educational level with the aphasic individuals. Two control groups were included: the first one included 43 subjects (22 males and 21 females, mean age: 53.4) with moderate–high school attendance (>6 years, mean school attendance: 10.4), and the second group included 33 subjects (21 males and 12 females; mean age: 67.9) with low school attendance (≤5 years mean school attendance: 4.5). Subjects with a history of neurological impairment and developmental linguistic disorders were not included in the control sample.

The study was conducted in accordance with the Declaration of Helsinki (1964) and was approved by the Ethical Committee.

## 4. Analysis of Lesions

Lesion sites were classified on the basis of neuroradiological and clinical evidence of the cerebral arterial territory involved. In particular, the Oxford Community Stroke Project (OCSP; [27]) classification was adopted. Most of the patients (69%) presented partial anterior circulation infarcts (PACI), 8% had posterior circulation infarcts (POCI), 6% had total anterior circulation infarcts (TACI), and very few (1%) had lacunar lesions (LACI). Note that neuroimaging exams were performed to identify lesion location, but further information such as lesion volumes could not be established.

## 5. Materials

The CoBaGa (Cognitive Test Battery for Global Aphasia; [28, 29]) was used to assess cognitive functions. This is a test battery that is suitable for patients with severe aphasia because it requires only manipulative answers and not verbal responses. The CoBaGa is made up of five subtests that evaluate the following cognitive functions: attention, executive functions, logical reasoning, memory, visual-auditory recognition, and visual-spatial ability. The score of each subtest is the sum of the scores obtained on the various items. There is no time limit. A more detailed description of the items included in each subtest can be found in Appendix A.

The CoBaGa is a reliable battery; it has a good test-retest correlation one month after it has been administered (*r* = 0.71) and good internal consistency (Cronbach's *α* is 0.80 in all tests and 0.73 and 0.9 in different subsets) and discriminant validity (i.e., it can discriminate patients with aphasia from healthy subjects, *p* < 0.0001, and patients with aphasia from neurological patients without aphasia, *p* < 0.05). Moreover, the CoBaGa has good convergent validity with other etero-valutative instruments (with cognitive scores of Functional Independence Measure (FIM; [30]): *r* Pearson (Pearson's*r*) = 0.72, *p* < 0.001) as well as good divergent validity (with FIM motor score: *r* Pearson (Pearson's*r*) = 0.32, *p* = ns). The CoBaGa also has proven sensitive in detecting follow-up changes in performance (at least *p* < 0.05).

Patients' cognitive ability was tested with the CoBaGa (see Appendix A) and with two different linguistic tests to ensure the generalization of results. In particular, 63 patients were examined with the Aachener Aphasia Test (AAT; [31]) and 111 patients with the Examination of Language Test [32]. The Token Test [26] was used to analyze severity of aphasia. Only 15 patients were administered another language test; due to the small sample, these data were not analyzed. The following language abilities were investigated: repetition, reading-spelling, naming, and oral and written comprehension. A more complete description of the subtests used in the two language batteries can be found in Appendix B.

## 6. Procedure

All patients consecutively admitted to the Neuropsychological Unit of the Department of Neurological Rehabilitation, Salvatore Maugeri IRCCS Foundation, Cassano delle Murge (Bari, Italy), from 2005 to 2010 participated in the study. Patients and their caregivers were informed about the aims of the study and gave their consent to participate. To avoid distraction, patients were evaluated individually in a quiet room. Testing was interrupted if a patient showed any sign of tiredness. Before the tests were administered, information was gathered concerning patients' clinical history and previous abilities. Any human data included in this manuscript was obtained in compliance with the Declaration of Helsinki.

## 7. Data Analysis

A cluster analysis was performed on accuracy scores of the different subtests of the CoBaGa. A hierarchical cluster analysis was performed preliminarily to determine the number of clusters in the examined population. Subsequently, a K-means cluster analysis was performed to optimize the assignment of patients to the clusters.

A one-way analysis of variance (ANOVA) was performed to determine whether the clusters of the patients identified differed as to sociodemographic and clinical variables such as age (in months), time from stroke, and years of schooling. Significant differences were examined with the post hoc Tukey test. Analysis of *χ*^2^ was performed to verify gender distribution in the groups.

An ANCOVA analysis was used to determine whether the groups performed differently on the CoBaGa subsets. The dependent variable was the accuracy percentage on each CoBaGa subset. The independent variable was the cluster membership of the subjects. Significant sociodemographic variables that emerged from previous analyses were used as covariates in the ANCOVA.

Moreover, to better characterize the cognitive impairment in each group, an ANOVA was performed with the type of subset (i.e., five levels, corresponding to the five cognitive functions examined) as independent variable and the subset mean percentages of accuracy for each cluster as dependent variables. The significant effects were explored with a post hoc Tukey test.

Finally, for each group, the number of patients who performed pathologically was computed for each subset. Severity of impairment was also evaluated. In particular, performances 2 SD lower than the mean of the control groups paired for school attendance (low or high school attendance) were considered pathological. In patients who performed pathologically, the severity of the deficit was evaluated on the basis of the performance of the whole group of patients [13] with comparable amount of school attendance (high versus low). In particular, according to Kalbe et al. [13], patients who performed below the 30th percentile of the whole group distribution scores had a severe deficit, patients with performances ranging from the 31st to the 60th percentile had moderate impairment, and patients whose performances were higher than the 61st percentile had mild deficits. In each group identified by cluster analysis, the prevalence of severe, moderate, and mild deficits or normal performance on each cognitive function was explored by the *χ*^2^ tests.

An ANCOVA was performed on the accuracy scores of both the EoL and the AAT tests to verify whether subjects who belonged to the three clusters differed as to accuracy on the linguistic tests. Significant sociodemographic variables were used as covariates. Interactions were explored by planned comparisons.

Furthermore, the linguistic skills able to predict the total score on the CoBaGA were also examined separately for the AAT and the EoL score. In particular, regression analyses were performed with the total score on the CoBaGa as dependent variable and oral and written comprehension, naming, reading-spelling, and repetition as independent variables. Analyses were replicated also controlling for the effect of significant sociodemographic variables. In this case, the significant sociodemographic variables were entered first as predictors and after the other language scores.

## 8. Results

### 8.1. Profiles of Cognitive Deficits in Aphasic Patients

The cluster analysis showed that there were three subgroups of patients with different cognitive profiles. The first group included 34% of the patients (65 patients), the second group 40% (75 patients), and the third group 26% (49 patients). Demographic and clinical characteristics of the three groups are presented in Table 1.

The analysis of variance revealed that the three groups of patients were comparable for mean age and time from stroke but differed for years of schooling (*F*_(2,182)_ = 8.52, *p* < 0.0001). In particular, the first group had a significantly higher educational level than the second (*p* < 0.001) and the third (*p* < 0.001) groups. No gender differences were observed in the three groups (all *χ*^2^ n.s.).

**Figure 1:**
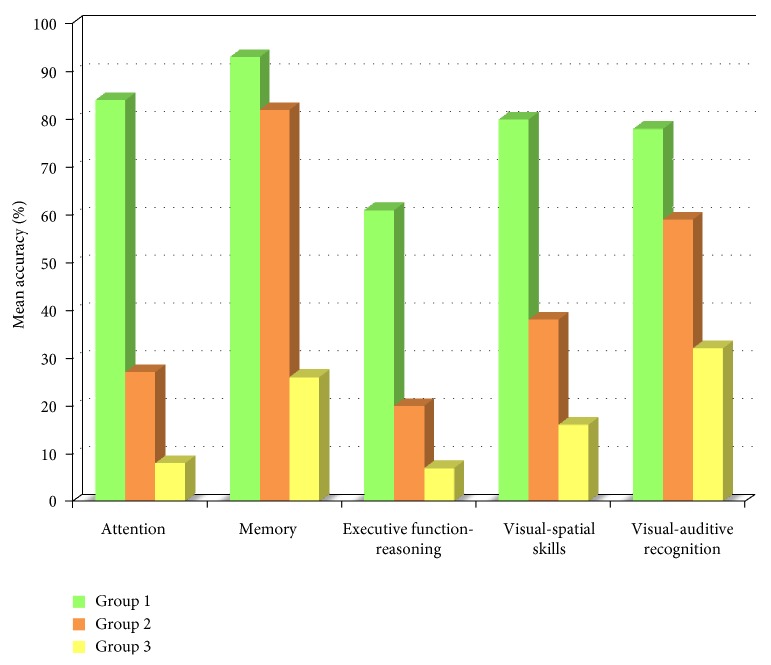
Mean accuracy of the three cognitive profiles on the CoBaGa subsets.

Figure 1 shows mean percentages of accuracy for each subset in the three subgroups of patients. According to the ANCOVA, the three groups had significantly different performances in all subsets of the CoBaGa (*p* < 0.0001 in all comparisons) also when the level of school attendance of the patients was taken into account. The first group had higher percentages of accuracy than the second and the third groups for all cognitive functions. The second group had intermediate percentages of accuracy, and the third group had the lowest percentages of accuracy in all subtests, suggesting severe cognitive impairment.

In each group, a significant difference emerged for subtest accuracy (group 1: *F*_(4,256)_ = 40.7, *p* < 0.0001; group 2: *F*_(4,296)_ = 135.3, *p* < 0.0001; group 3: *F*_(4,192)_ = 27, *p* < 0.0001). An examination of subtest means showed that the first group had the highest percentages of accuracy in the memory test (93%, *p* < 0.01 compared with other subsets) and the lowest percentages of accuracy in the executive functions and logical reasoning subsets (61%, *p* < 0.0001 compared with other subsets). The accuracy of this group in attention, visual-spatial ability, and visual-auditory recognition tests was comparable, with 80% accuracy. Subtest comparisons were also significant in the second (at least *p* < 0.05) and in the third group (*p* < 0.05). The second group was characterized by high performance in the memory test (82% of accuracy), moderate performance in visual-auditory recognition (59%), and low performance in visual-spatial ability (38%), attention (27%), and executive functions-logical reasoning (20%). The third group was characterized by generally low percentages of accuracy, that is, 7% for attention and executive functions-logical reasoning, 16% for visual-spatial ability, 25% for memory, and 33% for visual-auditory recognition.

**Figure 2:**
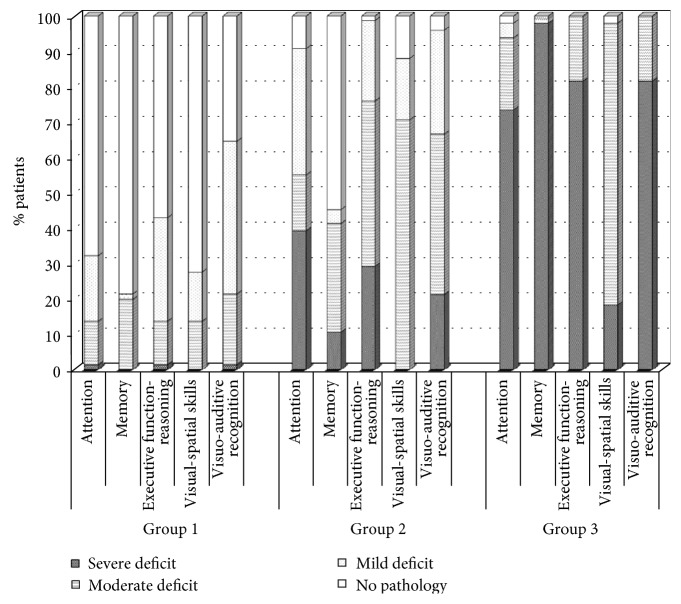
Percentage of patients in each group with deficits in cognitive functions differentiated by severity of impairment.

Figure 2 and Table 2 present for each group the percentages of patients with severe, moderate, and mild pathology in each subtest. As observed in Figure 2, the three profiles differed progressively for severity of cognitive impairment. In fact, for all cognitive functions studied, the percentage of impaired patients was lower in the first group than in the second (at least *p* < 0.01) and the third (at least *p* < 0.0001) groups. Conversely, the percentage of patients with severe cognitive deficits increased progressively from the first to the third group (at least *p* < 0.01) in each subtest, with the exception of visual-spatial impairment (patients with this deficit were not observed in the first and the second group).

In particular, in the first group, the percentages of patients with nonpathological conditions were significantly higher than those of patients with mild, moderate, and severe deficits (at least *p* < 0.05 for all comparisons). The percentages of nonpathological patients were 67% for attention, 78% for memory, 56% for executive functions-logical reasoning, and 72% for visual-spatial ability. The percentages of nonpathological patients were 35.4% only for the visual-auditory recognition subset; in fact, 43.1% and 20% of patients had mild or moderate deficits, respectively. The percentages of patients with severe deficits in the first group were very low (1.5% for attention, executive functions-logical reasoning, and visuo-acoustic recognition) or zero (in memory and visual-spatial ability) and were significantly lower than the percentages of patients with mild and moderate deficits (at least *p* < 0.05 for all comparisons). Regarding the comparison between moderate and mild deficits in the first group, moderate impairment of memory (*p* < 0.001) and mild deficits in executive functions and logical reasoning (*p* < 0.05) were those most frequently observed. No significant differences were observed between patients with mild and moderate impairments of attention and visual-spatial ability.

The second group of patients showed heterogeneous performances. A moderate deficit of visual-spatial ability (for 70.7% of patients; at least *p* < 0.0001), executive functions-logical reasoning (for 47% of patients; at least *p* < 0.001), and visual-auditory recognition (for 45% of patients; at least *p* < 0.001) prevailed in this group. Memory abilities were normal in 55% of the patients and moderately impaired in 31% of patients; 40% and 36% of patients in this group demonstrated, respectively, severe and mild impairment of attention.

According to Figure 2, in the third group, most patients had severe deficits in all subtests (from 73.5% to 98% of patients; *p* < 0.0001 in all comparisons) except for visual-spatial ability. Moderate deficits were less frequently observed (from 2% to 20.4%; *p* < 0.001 in all comparisons) in this group. Moderate impairments were frequently observed only for visual-spatial ability (79.6%), and severe deficits were rare (18.4%; *χ*^2^ = 18.8; *p* < 0.0001). The percentage of patients who performed normally or had mild impairments was low (from 0% to 4.1%).

### 8.2. Brief Summary of Results

Patients with severe aphasia can be divided into three groups according to their cognitive profile. In the first group, most patients' intellectual functions were spared and a high percentage of patients had no pathology. The second group was more heterogeneous and performances were generally worse than in the first group. The third group primarily included patients with severely impaired performance in all cognitive functions investigated.

In particular, the first group showed high mean accuracy for all cognitive functions studied. In fact, this group consisted mainly of patients without cognitive impairment who had visual-auditory recognition abilities. One-third of the patients in this group had no recognition deficits, but two-thirds showed mild–moderate impairment.

The second group of patients was the most heterogeneous. The best mean performance of this group was observed on the memory subtest. In fact, a large percentage of patients in this group displayed no memory deficits. Two-thirds of the patients showed moderately impaired visual-spatial ability, but none suffered from a severe deficit. Almost all patients in this group had deficits in executive functions-logical reasoning and visual-auditory recognition. In particular, half of the patients demonstrated moderate deficits of these functions and the rest showed mild or severe impairment. The attention deficit of this group was mostly severe or mild.

The third group had very low mean accuracy in all subtest of the CoBaGa. This group was composed mainly by patients with severe deficits in all cognitive functions except for visual-spatial ability. In fact, most aphasic patients in this group had moderately impaired visual-spatial skills.

These three subpopulations of patients did not differ for mean age and time from stroke. Therefore, it seems that these variables do not influence cognitive functioning. The three groups were different regarding level of school attendance. In particular, the group with the best cognitive efficiency was characterized by the highest level of school attendance. We can suppose that a high level of school attendance served as a protective factor for cognitive functions. These results contradict the results reported by Helm-Estabrooks et al. [23]. According to these authors, cognitive functions were not influenced by age and level of school attendance.

### 8.3. Cognitive Deficits and Language Skills

**Figure 3:**
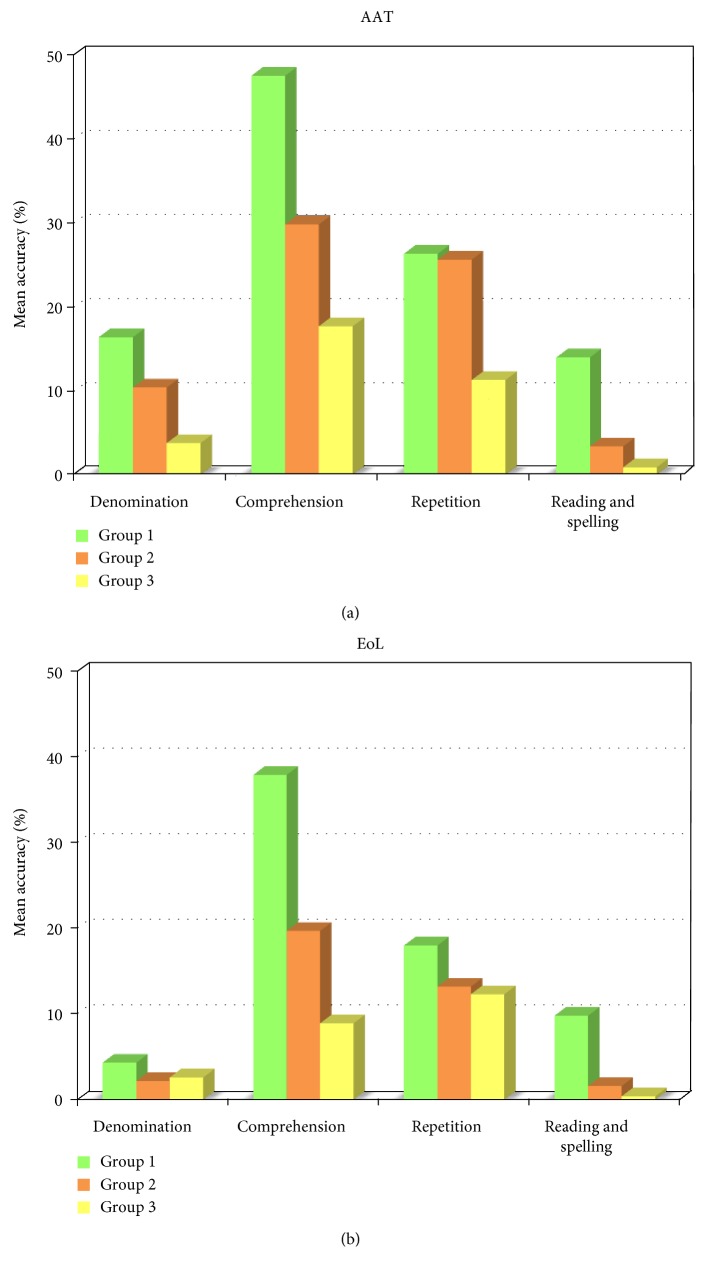
Mean accuracy of linguistic ability in the three groups according to AAT (a) and EoL (b).

The previous analysis revealed the presence of three different cognitive profiles in patients with severe aphasia. The aim of this second set of analyses was to verify whether the groups clustered on the basis of different cognitive profiles were characterized by different residual linguistic abilities. The performance of the three groups on the EoL and AAT is presented in Figure 3.

According to the ANCOVA, the groups were comparable for repetition and naming, but differed for reading-spelling abilities (AAT: *F*_(2, 59)_ = 14.98, *p* < 0.0001; EoL: *F*_(2,106)_ = 19.38, *p* < 0.0001) and oral-written comprehension (AAT: *F*_(2, 59)_ = 4.4, *p* < 0.05; EoL: *F*_(2,106)_ = 22.9, *p* < 0.0001). The first group was significantly more accurate in reading and spelling than the second (AAT: *F*_(2, 59)_ = 13.05, *p* < 0.001; EoL: *F*_(2,106)_ = 28.7, *p* < 0.0001) and the third (AAT: *F*_(2, 59)_ = 26.17, *p* < 0.0001; EoL: *F*_(2,106)_ = 32.56, *p* < 0.0001). In fact, the first group's reading-spelling accuracy was 14% on the AAT and 10% on the EoL, whereas the second (AAT: 3%, EoL: 1%) and third (AAT: 2%, EoL: 0%) groups were characterized by lower accuracy. The second and third groups were similar with regard to reading-spelling abilities. The three groups performed differently in oral and written comprehension (at least *p* < 0.01). In fact, in this test, the first group showed higher accuracy (AAT: 48%, EoL: 38%) compared to the second group (AAT: 30%, EoL: 20%) and especially the third group (AAT: 18%, EoL: 9%).

The regression analysis performed on the scores of the AAT showed that linguistic skills explain 43% of the variance in cognitive performance. Significant predictors were naming (*β* = −0.41, *t* = −2.06, *p* < 0.05), comprehension (*β* = 0.65, *t* = 5.72, *p* < 0.0001), and reading and spelling skills (*β* = 0.27, *t* = 1.97, *p* < 0.05). When years of school attendance were added to the analysis, the variance explained by the model was 45%. In this case, comprehension was a significant predictor (*β* = 0.64, *t* = 5.43, *p* < 0.0001) and reading and spelling approached significance (*β* = 0.25, *t* = 1.76, *p* = 0.08); however, naming was still not significant (*β* = −0.35, *t* = 1.66, *p* = 0.10). Years of school attendance was not significant. Repetition skill did not predict the CoBaGa performance in either analysis.

The regression analysis performed on the EoL scores demonstrated that language skills explain 60% of the variance on the CoBaGa test. Significant predictors were naming (*β* = −0.27, *t* = −2.44, *p* < 0.05), comprehension (*β* = 0.72, *t* = 8.65, *p* < 0.0001), and reading and spelling skills (*β* = 0.19, *t* = 2.15, *p* < 0.05). When years of school attendance were added to the analysis, the variance explained by the model was 63%. In this case, significant predictors were naming (*β* = −0.24, *t* = −2.19, *p* < 0.01) and comprehension (*β* = 0.69, *t* = 8.58, *p* < 0.0001) as well as years of school attendance (*β* = 0.19, *t* = 3.10, *p* < 0.01). Reading and spelling skills were still not significant (*β* = −0.14, *t* = 1.66, *p* = 0.10). Repetition skill did not predict the CoBaGa performance in either analysis.

### 8.4. Brief Summary of Results

The three groups identified on the basis of cognitive abilities also had different residual linguistic skills, in particular they differed for comprehension, reading, and spelling performance. The results were confirmed independently of the linguistic test used. Therefore, it seems that comprehension and reading-spelling skills were the linguistic abilities most linked to general cognitive functioning.

Regarding linguistic abilities that differed in the three groups, it seems that there were differences in severity of linguistic impairment between groups. Therefore, severity of the linguistic deficit seems to be connected to severity of the cognitive impairment. In fact, the group with mild cognitive deficits (group 1) demonstrated less marked difficulty in reading-spelling and comprehension than the second and especially the third group. In fact, the third group was characterized by the most severe deficits in both linguistic and cognitive abilities. We can suppose that linguistic difficulties and patients' general cognitive functioning were strictly related.

The present study also shows that it is possible to predict the cognitive profile of patients with severe aphasia on the basis of linguistic impairments, and in particular on the basis of naming, comprehension, and reading and spelling skills. This result is in accordance with the results of Kalbe et al. [13] and Hinckley and Nash [25], which support the existence of a relationship between cognitive and linguistic deficits. In fact, the present results do not support the hypothesis [6, 24] of total independence between the two impairments and the impossibility of predicting linguistic deficits on the basis of cognitive ability. The small number of subjects examined by Van Mourik et al. [24] and Helm-Estabrooks [6] may be responsible for the discordant results.

## 9. Study 2

Twenty patients were examined longitudinally pre and post speech therapy to investigate whether, by the end of therapy, language treatment also improves cognitive skills and which cognitive abilities are most likely to recover after speech therapy.

## 10. Participants

The 20 patients (11 females and 9 males) in study 1 also participated in study 2. They were tested at the end of speech therapy, which lasted about 4 months (SD = 2). They were all right handed and with global aphasia. The mean age of the sample was 65 years (SD: ±7.8) and the mean educational level was 6.6 (SD: ±3) years. The patients were examined 101 (SD: ±81) days after they had experienced a stroke. Almost all of them had partial anterior circulation infarcts (PACI), and two had total anterior circulation infarcts (TACI), following the Bamford et al. [27] classification. Among all of them, 11 patients belong to the 3rd group and 9 to the 2nd group in study 1. Language therapy was focused on both oral and written comprehension and production, as well as on rehabilitation of articulatory difficulty.

## 11. Materials

All participants performed the CoBaGa and EoL both pre and post test.

## 12. Procedure

Same as that in study 1.

## 13. Data Analysis

Preliminarily, we checked whether speech therapy led to improved language skills, as expected. In particular, an ANOVA was performed with treatment (pre- versus post-) and language skills (comprehension, naming, repetition, reading, and spelling) as repeated measures.

With respect to the specific aims of this study, a second ANOVA was performed on the CoBaGa scores to determine whether speech therapy also resulted in detectable improvements in cognitive performance. In particular, treatment (pre- versus post-) and type of subset (5 levels: corresponding to the five cognitive functions examined) were entered as repeated measures.

All analyses were replicated also controlling for years of education and duration of speech therapy (in days) to assess whether these variables mediated the relation between cognition and language. In particular, in the ANOVAs, these variables were entered as covariates.

## 14. Results

### 14.1. Effect of Speech Therapy on Language Skills

As expected, speech therapy led to improved language performance. In fact, the ANOVAs showed the significance of the main effects of the rehabilitation (*F*_(1, 18)_ = 10.16, *p* < 0.01) and language domain (*F*_(3, 54)_ = 17.05, *p* < 0.0001), indicating that accuracy improved from 13.5% to 20.0% and that comprehension and repetition skills were more impaired than naming and reading-spelling skills (5.1% and 7.2% versus 33.8% and 20.6%, resp., at least *p* < 0.01). Also, the rehabilitation by language domain interaction was significant (*F*_(3, 54)_ = 5.16, *p* < 0.01), indicating a significant improvement in performance in the posttest respect to the pretest only for comprehension (reduction of errors of 12.3%, *p* < 0.0001) and repetition (reduction of errors of 6.1%, *p* < 0.05), but not for naming (3.3%) and reading and spelling skills (4.0%).

When education and speech therapy duration were added as covariates, only the main effect of rehabilitation was still significant (*F*_(1, 16)_ = 8.72, *p* < 0.01); in fact, the language domain (*F*_(3, 48)_ = 1.50, n.s.) and the rehabilitation by language domain (*F*_(3, 48)_ = 0.97, n.s.) interactions were no longer significant, indicating that all areas of language improved with language rehabilitation if years of schooling and therapy duration were taken into account. Neither covariate was significant (Fs < 1).

### 14.2. Effect of Speech Therapy on Cognitive Skills

The ANOVAs on cognitive skills pre and post speech therapy revealed the significance of the main effect of rehabilitation (*F*_(1, 24)_ = 23.29, *p* < 0.0001) and cognitive domain (*F*_(4, 96)_ = 36.29, *p* < 0.0001), indicating that accuracy improved from 24.9% to 33.1% and that memory and visual-spatial skills were more impaired with respect to attention, executive function and visuo-acoustic recognition (5.9% and 15.0% versus 38.7%, 37.8% and 47.8%, resp., at least *p* < 0.0001). The rehabilitation by cognitive domain interaction was also significant (*F*_(4, 96)_ = 7.93, *p* < 0.0001), indicating a significant improvement in performance only for attention (improvement of 21.2%, *p* < 0.0001) and reasoning/executive function (improvement = 12.6%, *p* < 0.01) after speech therapy; memory (improvement = 0.9%), visual-spatial ability (improvement = 2.0%), and visuo-acoustic recognition (improvement = 4.3%) did not differ pre and post test.

When covariates were added to the analysis, the results did not change: both the main effect of rehabilitation (*F*_(1, 24)_ = 21.53, *p* < 0.0001) and cognitive domain (*F*_(4, 96)_ = 33.90, *p* < 0.0001) were still significant, as well as the rehabilitation by cognitive domain (*F*_(4, 96)_ = 6.58, *p* < 0.0001) interaction. Both covariates were nonsignificant (Fs < 1).

### 14.3. Brief Summary of Results

As expected, speech therapy produced a significant improvement in each linguistic domain. It also improved cognitive skills and, in particular, attention and reasoning/executive functions.

## 15. General Discussion

The present study indicates that in subjects with severe aphasia, it is possible to identify subgroups of patients with different profiles of cognitive impairment. Here, three subgroups were identified. The first was characterized by relatively spared cognitive abilities but visual-auditory recognition deficits. These patients seemed to have no cognitive impairment but had linguistic and recognition difficulties. The second group of aphasic patients presented with spared memory and moderate deficits in other cognitive functions. This cognitive profile was accompanied by mild deficits of attention in some patients and very severe deficits in others. The third cognitive profile was characterized by the lowest percentages of accuracy in all subtests, indicating severe and diffuse cognitive deficits. Our finding of three different cognitive profiles in global aphasic patients confirms previous observations by Van Mourik et al. [24] in a large sample of patients. As Van Mourik et al. [24] suggested, groups with different profiles may have different outcomes in rehabilitation training. This hypothesis should be further investigated in subsequent studies.

The present study also showed that groups with different cognitive profiles also have different reading-spelling skills and oral-written comprehension abilities. In particular, according to previous studies [21, 25], patients with more severe cognitive impairment also have more severe linguistic deficits. Moreover, the link between language impairment and general cognitive functioning is also supported by the finding that it is possible to predict with a certain degree of accuracy the cognitive profile of patients with severe aphasia on the basis of their linguistic impairment. In this framework, reading and spelling abilities, naming, and oral-written comprehension skills have a crucial role. The latter language abilities are probably those most affected by cognitive impairment. The nature of this relationship, as well as the specific type of cognitive deficit that compromises reading-spelling and comprehension abilities, requires further investigation.

A strong relationship between linguistic deficits and cognitive skills was also found longitudinally in a study that examined patients' pre and post speech therapy. Note that speech therapy produces a nonspecific improvement in cognitive skills that goes beyond language recovery. In fact, we found that speech therapy not only improves language skills but also attention, reasoning, and executive functions. Thus, it seems that these abilities were involved in the speech therapy and were improved. On the other hand, most studies (e.g., [33]) agree about the importance of executive functions, working memory, and attention for the efficacy of language therapy. As suggested by Fonseca et al. [7], attention and nonverbal memory “are two abilities that might be systematically evaluated as baseline measures that might affect the success of speech rehabilitation.” (p. 11)

In any case, the relationship between language and other cognitive domains is still controversial and different hypotheses have been proposed to explain it [34]. For example, in the 1800s, Finkelnburg [35] hypothesized that the disruption of preverbal symbolic activities caused the verbal and nonverbal cognitive disorders in aphasic patients. Trousseau [36] considered language very important for the development of thought and proposed that severe language disorders might lead to impairment of both verbal and nonverbal cognitive skills. Later, Goldstein [37] proposed that language is not only a means of communicating thoughts but is also important for its development. Davis [38] defined cognition as an information processing skill; specifically, because language uses information processing, it may be embedded in cognition.

In more recent years, Jefferies and Lambon Ralph [39] proposed the “semantic hub” hypothesis to explain the “unexpected brain-language relationships in aphasia” [40]. These authors assumed that the deficits of aphasic patients are due to a preverbal conceptual disorder which cannot be attributed to a loss of semantic representations but rather to a deficit in their controlled retrieval. The cognitive deficit is due to a control deficit involved in the selection and activation of conceptual representations: these mechanism of semantic control flexibility activate information by means of the underlying amodal concept and focus attention on particular features of concepts (while ignoring others) to produce task/context-appropriate behaviour. In this vein, aphasic patients have difficulty in controlling semantic representations appropriately and in working flexibly with the knowledge they have retained. This deficit in cognitive control is associated with executive function impairment (i.e., in the left inferior prefrontal cortex). In this vein, the relationship between language and cognition is mediated by executive functions.

Other hypotheses considered the role of executive functions [13, 41], short-term memory [42, 43], or attentional resources [9, 44] in negatively affecting language deficits. According to an attentional hypothesis, syntactic processing deficits in aphasia can be explained by a deficit of resource capacity or a reduced ability to allocate attentional resources [45]. McNeil et al. [3] also proposed an “integrated attention theory of aphasia.” According to these authors, there is a relationship among attention, arousal, and language processing and individuals with aphasia have a deficit in allocating attentional resources.

Cahana-Amitay and Albert [46] incorporated nonlinguistic functions into language models and hypothesized the existence of “neural multifunctionality” in which a constant and dynamic interaction exists among neural networks subserving cognitive, affective, and praxic functions with neural networks specialized for lexical retrieval, sentence comprehension, and discourse processing, giving rise to language.

According to other authors, language and cognition are not strictly related. For example, Hauser et al. [47] argued that language is an abstract linguistic computational system which is independent of other systems it interacts with and establishes interfaces. In fact, the presence of aphasia does not necessarily produce other neuropsychological impairments [48] and cognitive deficits in aphasic patients are not always correlated with language impairment (e.g., [49, 50]). Recently, some authors [51] provided evidence from neuroimaging and neurological data that despite global aphasic patients' near-total loss of language, they are able to perform some nonlinguistic tasks such as arithmetic, storing information in working memory, inhibiting prepotent responses or listening music. The authors concluded that many aspects of cognition engage distinct brain regions which do not necessarily depend on language. On the other hand, Fonseca et al. [7] reported that patients with aphasia always perform similarly to patients with brain damage without aphasia. This indicates that some of the impairments of aphasic patients are not secondary to language impairment but to brain dysfunction per se. As highlighted by Seniów et al. [52], evidence of aphasia is not necessarily associated with impairment of other cognitive functions, suggesting that these deficits may be independent of one another.

The present study highlights that patients with severe aphasia are heterogeneous with regard to cognitive impairment, which ranges from spared to severely impaired cognitive function. In any case, in this study, we found a strong relationship between language impairment and general cognitive functioning: patients with more severe cognitive impairment also had more severe linguistic deficits. Note that in this study, the relationship between linguistic deficits and cognitive abilities was evaluated in a population of patients with severe aphasia. We do not know whether the present results can be generalized to the entire population of patients with aphasia. It might be interesting to repeat this study in a population of patients with less severe aphasia to examine the relationship between linguistic impairment and cognitive abilities and to identify cognitive profiles among these patients. Moreover, the population of the present study was characterized by severe aphasia and both language comprehension and production deficits. It would be interesting to know whether different profiles of cognitive impairment in patients with less severe aphasia are related to different syndromes of aphasia and selective deficits of linguistic comprehension/production. Moreover, we do not know the role of other variables that might affect patients' cognitive profile, such as premorbid IQ and other factors that might affect cognitive performance.

In light of the present results, we can affirm the importance of assessing cognitive functions as well as linguistic deficits in aphasic patients. A correct assessment of cognitive abilities and comprehension of lost and preserved functions might be useful in programming individualized rehabilitation training. Several studies [53–55] found a reduction of linguistic deficits after rehabilitative training for attention, memory, visual perception, or executive function-problem solving in aphasic patients who did not benefit from speech and language treatment. This suggests the greater advantage of combined rehabilitation for both language impairment and cognitive deficits in aphasic patients.

## Figures and Tables

**Table 1:** Demographic and clinical variables of the three groups of patients with different cognitive profiles.

Group	Number of subjects	Sex	Age (years)	School attendance (years)	Time from disease (months)	Token test accuracy (*N* = 36)
1	65 (34%)	23 F, 42 M	63.8 (SD: 10.9)	7.9 (SD: 4.6)	127 (SD: 157)	8.8 (SD: 4.7)
2	75 (40%)	35 F, 40 M	65.7 (SD: 11.5)	5.5 (SD: 3.2)	121 (SD: 190)	5.9 (SD: 4.3)
3	49 (26%)	20 F, 29 M	68.7 (SD: 12.3)	5.2 (SD: 3.9)	135 (SD: 196)	4 (SD: 4.1)

F: female; M: male; for the age variable, school attendance, time from disease, and Token test accuracy, means and standard deviations (SD) of each group are reported.

**Table 2:** Percentage of patients in each group with a deficit in the cognitive function was investigated and computed for severity of the impairment.

Severity of cognitive deficit	Attention			Memory			Executive functions-logical reasoning			Visual-spatial ability			Visual-auditory recognition		
	Group 1	Group 2	Group 3	Group 1	Group 2	Group 3	Group 1	Group 2	Group 3	Group 1	Group 2	Group 3	Group 1	Group 2	Group 3
Severe	1.5%	39.7%	73.5%	0%	10.7%	98%	1.5%	29.3%	81.6%	0%	0%	18.4%	1.5%	21.3%	81.6%
Moderate	12.3%	16%	20.4%	20%	30.7%	2%	12.3%	46.7%	18.4%	13.8%	70.7%	79.6%	20%	45.3%	18.4%
Mild	18.5%	36%	4.1%	1.5%	3.9%	0%	29.2%	22.7%	0%	13.9%	17.3%	0%	43.1%	29.3%	0%
No deficit	67.7%	9.3%	2%	78.5%	54.7%	0%	56.9%	1.3%	0%	72.3%	12%	2%	35.4%	4%	0%

## Appendix

### A. Tests Included in Each Subtest of the CoBaGa [28]

#### 1. Attention

This subset included letter cancellation [56], numbers [57], and the Toulouse-Pieron test [58].

In the first test, patients were asked to cross out all the “A's” in a series of letters arranged in 17 lines. The maximum score was 60 (1 point for each letter identified).

In the second test, the numbers from 0 to 9 were arranged in 13 lines. The patients were asked to cross out all the number “5's.” The maximum score was 14, corresponding to the total number of target stimuli.

In the Toulouse-Pieron test, the patients were instructed to identify and cancel a specific symbol (one square and two segments oriented in a particular way inside this square). Different symbols were arranged in 10 lines. The maximum score was 13.

Accuracy on this subset was the total number of correct answers minus the number of false alarms.

#### 2. Executive Functions and Logical Reasoning

This subset consisted of a modified version of Raven's Coloured Progressive Matrices [59] and some WAIS-R tests [60]. In particular, it included the construction of a human figure, drawing with cubes, association of symbols and numbers (reduced version), and two sequence ordering tests of the WAIS-R.

Raven's Coloured Progressive Matrices consist of 36 items of increasing difficulty. The subject has to choose the one item from 6 different alternatives that completes the figure according to an exact logic. In the present study, the alternatives were located one under the other on the left side of the page to limit the effects of difficulty with visual-spatial exploration. The score was calculated as the sum of correct answers. The maximum score was 36.

In the construction of a human figure test, a shape of the human body was presented on one page along with 5 cards showing a head, trunk, and four limbs. Patients had to recompose the dummy using the 5 cards and the shape of the human body. The maximum score was 5 and corresponded to the number of correct positions of the cards.

Drawing with cubes involved reproducing 6 figures presented on the pages using 4 colored cubes. The maximum score was 24; it was the correct combination of cubes to construct the figure.

In the test of association of symbols and numbers, the patients were shown numbers from 1 to 9 that were associated with different symbols located under each number. Forty numbers were presented below in random order. The patients had to reproduce the correct symbol under each number, as demonstrated in the example. The maximum score (i.e., 40) corresponded to the correct number of associations.

In the sequence ordering test, the patients had to correctly order a series of cards based on the temporal sequence of events. Two different situations were used. The first sequence represented the blooming of a plant (from bud to flower, 3 cards). The second sequence represented the construction of a house (starting from bricks and arriving at a complete house in 4 cards). The score was the sum of the number of cards located correctly in each sequence. The maximum score was 7.

#### 3. Memory

This subset consisted of a visual memory test of faces and objects.

The patients were shown four faces and were told to remember them. Subsequently, the same faces were shown to the patients in random order together with 4 distractors. The patients were asked to recognize the faces shown before. The score was the number of correct answers.

The same procedure and score calculation were used in the memory test for objects. This probe required recognizing 4 objects out of 8 figures.

#### 4. Visual-Auditory Recognition

This subset consisted of the following tests: recognition of unknown faces [61], identification of complex figures [62], and identification of colors, association of colors, association of figures, association of objects and colors, association of objects and figures, association of visually different objects and figures, and recognition of sounds and noise. In all tasks, one point was given for each correct item.

The test of recognition of unknown faces consisted of two parts. First, patients had to recognize the target face out of 6 faces in different conditions of illumination. In the second part of the test, the target face was similar to 3 out of 6 alternative faces proposed.

In the identification of complex figures test (included in the left table), one complex shape without sense (target figure) was shown, and on the right three different complex shapes were shown. Patients had to recognize the target shape among the three figures shown in the tables on the right. The maximum score on this test was 4.

In the identification of colors test, one table was presented that showed six colors. The examiner named each color separately. The patients had to identify the named colors.

The association of colors test was presented in one table that showed six colors and six colored cards. The patients had to put the cards on the table by matching the corresponding colors.

The association of figures test was presented in two tables with 7 figures. One table was used by the examiner; another table was given to the patient. The examiner indicated one figure and the patient had to indicate the corresponding figure on the table.

The test of association of objects and colors was presented in one table containing six colors. The examiner demonstrated 6 objects to the patient, who had to associate each object with a corresponding color. The score was the number of correct answers.

The test of association of objects and figures consisted of one table that presented 6 figures and 6 corresponding objects. The patient had to associate each object with the corresponding figure.

The test of association of visually different objects and figures was presented in the same table described in the previous test and included 6 slightly different objects.

The patients listened to sounds registered on an audiocassette in the recognition of sounds and noise test. Then, they had to indicate the correct answer out of three possibilities illustrated in three squares.

#### 5. Visual-Spatial Ability

To evaluate visual-spatial ability, we used one test of line orientation judgment [63] and one test of recognizing objects shown in unusual perspectives.

In the judgment of line orientation test, 10 tables with lines drawn at different inclinations were shown to the patients. They had to compare the lines drawn in the table with a model consisting of lines of all possible inclinations. The score was the total number of lines whose orientation was correctly recognized. The maximum score was 20.

In the recognition of objects constructed in unusual perspectives test, the patients had to identify a target object out of three alternative objects drawn in different perspectives.

### B. Language Tests

#### 1. Aachener Aphasia Test (AAT; [30])

Only subsets of repetition, written language, naming, and comprehension of AAT [30] were used in this study.

The repetition subtest consisted of repeating single sounds, words with progressively increasing difficulty and length, foreign words, composed words, and phrases. Each part included 10 items. From 0 to 3 points were assigned for each repeated item on the basis of the subject's performance. The complex score of the repetition subset varied from 0 to 150.

The evaluation of written language skills included three different probes: reading aloud, dictation for composition, and dictation by hand of words or phrases. In particular, in the dictation for composition patients had to compose printed words or parts of words to form the complex words and phrases pronounced by the examiner. Each part included 10 items. The score of each subset varied from 0 to 30 and the total score varied from 0 to 90.

The naming subtest consisted of four parts (i.e., naming of objects, colors, objects with names, and descriptions of simple figures). Each of the parts included 10 items. The score for each of the four parts varied from 0 to 30 and the total score varied from 0 to 120.

The comprehension subtest consisted of four parts. Each of the parts included 10 items examining, respectively, oral comprehension of isolated words, oral comprehension of phrases, comprehension of written words, and comprehension of written phrases. Patients were required to identify the target figure out of the four alternatives presented. The score for each of the four parts varied from 0 to 30, and the total score varied from 0 to 120.

#### 2. Examination of Language (EoL; [31])

Tests evaluating naming ability, oral and written comprehension, repetition, and reading-spelling of the EoL [31] were administered to the patients.

In the naming ability test, subjects had to indicate figures representing nouns and verbs and describe one figure or event (one day spent at the sea or in the mountains).

Comprehension ability was evaluated using tests of oral and written comprehension. The oral part consisted of one test of word comprehension and of semantically similar words and phrases. In the first two cases, 20 figures were presented to the patients and they had to indicate the specific figure named by the examiner. In the phrase comprehension test, patients had to carry out the examiner's orders. In the test of written comprehension, patients had to read semantically similar words and phrases and to indicate the corresponding figure or carry out the order they had read.

In the repetition test, patients had to repeat phonemes, words, nonwords, and phrases pronounced by the examiner.

Finally, reading-spelling ability was tested with the copying of written words and reading and spelling to dictation of words, nonwords, sounds, and phrases.

Mean percentages of accuracy were calculated for each subset.

## References

[1] Caramazza A., Zurif E. B. (1976). Dissociation of algorithmic and heuristic processes in language comprehension: evidence from aphasia. *Brain and Language*.

[2] McNeil M. R. (1981). Auditory comprehension in aphasia: a language deficit or reduced efficiency of processes supporting language?. *Clinical Aphasiology*.

[3] McNeil M. R., Odell K., Tseng C. H. (1991). Toward the integration of resource allocation into a general theory of aphasia. *Clinical Aphasiology*.

[4] McNeil M. R., Kimelman M. D. Z. (1986). Toward an integrative information processing structure of auditory comprehension and processing in adult aphasia. *Seminars in Speech and Language*.

[5] Kang E. K., Jeong H. S., Moon E. R., Lee J. Y., Lee K. J. (2016). Cognitive and language function in aphasic patients assessed with the Korean version of Mini-Mental Status Examination. *Annals of Rehabilitation Medicine*.

[6] Helm-Estabrooks N. (2002). Cognition and aphasia: a discussion and a study. *Journal of Communication Disorders*.

[7] Fonseca J., Ferreira J. J., Martins I. P. (2016). Cognitive performance in aphasia due to stroke: a systematic review. *International Journal on Disability and Human Development*.

[8] Gainotti G., Caltagirone C., Miceli G. (1978). Immediate visual-spatial memory in hemisphere-damaged patients: impairment of verbal coding and of perceptual processing. *Neuropsychologia*.

[9] Murray L. L. (1999). Attention and aphasia: theory, research and clinical implications. *Aphasiology*.

[10] Duffy J. R., Watkins L. B. (2004). The effect of response choice relatedness on pantomime and verbal recognition ability in aphasic patients. *Brain and Language*.

[11] Kertesz A., McCabe P. (1975). Intelligence and aphasia: Performance of aphasics on Raven’s coloured progressive matrices (RCPM). *Brain and Language*.

[12] Purdy M. (2002). Executive function ability in persons with aphasia. *Aphasiology*.

[13] Kalbe E., Reinhold N., Brand M., Kessler M. J. (2005). A new test battery to assess aphasic disturbances and associated cognitive dysfunctions - German normative data on the aphasia check list. *Journal of Clinical and Experimental Neuropsychology*.

[14] Albert M. L. (1998). Treatment of aphasia. *Archives of Neurology*.

[15] Murray L. L., Ballard K., Karcher L. (2004). Linguistic specific treatment: just for Broca’s aphasia?. *Aphasiology*.

[16] Goldenberg G., Dettmers H., Grothe C., Spatt J. (1994). Influence of linguistic and nonlinguistic capacities on spontaneous recovery of aphasia and on success of language therapy. *Aphasiology*.

[17] Bailey S., Powell G., Clark E. (1981). A note on intelligence and recovery from aphasia: the relationship between Raven’s Matrices Scores and change on the Schuell Aphasia Test. *British Journal of Disorders of Communication*.

[18] El Hachioui H., Visch-Brink E. G., Lingsma H. (2014). Nonlinguistic cognitive impairment in poststroke aphasia: a prospective study. *Neurorehabilitation and Neural Repair*.

[19] Lee B., Pyun S. B. (2014). Characteristics of cognitive impairment in patients with post-stroke aphasia. *Annals of Rehabilitation Medicine*.

[20] Bonini M. V., Radanovic M. (2015). Cognitive deficits in post-stroke aphasia. *Arquivos de Neuro-Psiquiatria*.

[21] Kauhanen M. L., Korpelainen J. T., Hiltunen P. (2000). Aphasia, depression, and non-verbal cognitive impairment in ischaemic stroke. *Cerebrovascular Diseases*.

[22] Helm-Estabrooks N. (2001). *Cognitive Linguistic Quick Test*.

[23] Helm-Estabrooks N., Bayles K., Ramage A., Bryant S. (1995). Relation between cognitive deficit and aphasia severity, age, and education: female versus males. *Brain and Language*.

[24] Van Mourik M., Verschaeve M., Boon P., Paquier P., Van Harskamp F. (1992). Cognition in global aphasia: indicators for therapy. *Aphasiology*.

[25] Hinckley J., Nash C. (2007). Cognitive assessment and aphasia severity. *Brain and Language*.

[26] De Renzi E., Faglioni P. (1978). Normative data and the screening power of a shortened version of the Token test. *Cortex*.

[27] Bamford J., Sandercock P., Dennis M., Burn J., Warlow C. (1991). Classification and natural history of clinically identifiable subtypes of cerebral infarction. *Lancet*.

[28] Marinelli C. V., Craca A., Colucci A. (2006). Evaluation of cognitive deficit in global aphasia. *Neurological Sciences*.

[29] Marinelli C. V., Craca A., Lograno C., Angelelli P. (2009). The influence of cognitive abilities on language deficits: a longitudinal study on patients with severe aphasia. *European Journal of Neurology*.

[30] Forer S., Granger C. V. (1987). *Functional Independence Measure*.

[31] Luzzatti C., Willmes K., De Bleser R. (1996). *Aachen Aphasie Test (AAT): Italian Version*.

[32] Ciurli P., Marangolo P., Basso A. (1996). *Esame del linguaggio*.

[33] Fillingham J. K., Sage K., Lambon Ralph M. (2006). The treatment of anomia using errorless learning to aphasic disorders: a review of theory and practice. *Neuropsychological Rehabilitation*.

[34] Gainotti G. (2014). Old and recent approaches to the problem of non-verbal conceptual disorders in aphasic patients. *Cortex*.

[35] Finkelnburg D. C. (1870). Niederrhenische Gesellschaft, Sitzung vom 21 Marz 1870 in Bonn. *Berliner Klinische Wochenschrift*.

[36] Trousseau A. (1865). *Clinique Médicale de l’Hotel Dieu de Paris*.

[37] Glodstein K. (1948). *Language and Language Disturbances*.

[38] Davis G. A., Peach R. K., Shapiro L. P. (2012). The cognition of language and communication. *Cognition and Acquired Language Disorders*.

[39] Jefferies E., Lambon Ralph M. A. (2006). Semantic impairment in stroke aphasia versus semantic dementia: a case-series comparison. *Brain*.

[40] Berthier M. L. (2001). Unexpected brain-language relationships in aphasia: evidence from transcortical sensory aphasia associated with frontal lobe lesions. *Aphasiology*.

[41] Borod J. C., Carper M., Goodglass H. (1982). WAIS performance IQ in aphasia as a function of auditory comprehension and constructional apraxia. *Cortex*.

[42] Beeson P. M., Bayles K. A., Rubens A. B., Kaszniak A. W. (1993). Memory impairment and executive control in individuals with stroke-induced aphasia. *Brain and Language*.

[43] Christensen S. C., Wright H. H. (2010). Verbal and non-verbal working memory in aphasia: what three n-back tasks reveal. *Aphasiology*.

[44] Fucetola R., Connor L. T., Strube M. J., Corbetta M. (2009). Unravelling non-verbal cognitive performance in acquired aphasia. *Aphasiology*.

[45] Murray L. L., Holland A. L., Beeson P. M. (1997). Auditory processing in individuals with mild aphasia: a study of resource allocation. *Journal of Speech, Language, and Hearing Research*.

[46] Cahana-Amitay D., Albert M. L. (2014). Brain and language: evidence for neural Multifunctionality. *Behavioural Neurology*.

[47] Hauser M. C., Chomsky N., Fitch T. (2002). The faculty of language: what is it, who has it, and how did it evolve?. *Science*.

[48] Archibald Z. M., Wepman J. M., Jones L. V. (1967). Nonverbal cognitive performance in aphasic and nonaphasic brain-damaged patients. *Cortex*.

[49] Basso A., De Renzi E., Faglioni P., Scotti G., Spinnler H. (1973). Neuropsychological evidence for the existence of cerebral areas critical to the performance of intelligence tasks. *Brain*.

[50] Basso A., Capitani E., Luzzatti C., Spinnler H. (1981). Intelligence and left hemisphere disease: the role of aphasia, apraxia and size of lesion. *Brain*.

[51] Fedorenko E., Varley R. (2016). Language and thought are not the same thing: evidence from neuroimaging and neurological patients. *Annals of new York Academy of Sciences*.

[52] Seniów J., Litwin M., Leśniak M. (2009). The relationship between non-linguistic cognitve deficits and language recovery in patients with aphasia. *Journal of Neurological Sciences*.

[53] Helm-Estabrooks N., Connor L. T., Albert M. L. (2000). Treating attention to improve auditory comprehension in aphasia. *Brain and Language*.

[54] Kohnert K. (2004). Cognitive and cognate-based treatments for bilingual aphasia: a case study. *Brain and Language*.

[55] Murray L. L., Keeton J. L., Karcher L. (2006). Treating attention in mild aphasia: evaluation of attention process training-II. *Journal of Communication Disorders*.

[56] Diller L., Ben Yishai Y., Gerstman L. J., Goodkin R., Gordow W., Weinberger J. (1974). *Studies in Recognition and Rehabiltation in Hemiplegia*.

[57] Spinnler H., Tognoni G. (1987). Standardizzazione e taratura italiana di tests neuropsicologici. *Italian Journal of Neurological Sciences*.

[58] Toulouse E. Y., Pieron H. (1972). *Toulouse-Pieron: prueba perceptiva y de atencion manual*.

[59] Raven J. C., Court J. H., Raven J. (1977). *Coloured Progressive Matrices*.

[60] Wechsler D. (1981). *Wechsler Adult Intelligence Scale-Revised*.

[61] Benton A. L., Hamsher K. D., Varney N. R., Spreen O. (1992). *Test di riconoscimento di volti ignoti- Italian version*.

[62] Angelini R., Grossi D. (1993). *La terapia razionale dei disordini costruttivi (Te.Ra.Di.C.)*.

[63] Benton A. L., Hamsher K. D., Varney N. R. (1990). *Test di giudizio di orientamento di linee- Italian version*.

